# Examination of *Ureaplasma urealyticum* and *Mycoplasma hominis* in 4082 Chinese patients

**DOI:** 10.1590/1414-431X202010099

**Published:** 2020-11-27

**Authors:** Wei-wei Zheng, Wen-jing Zhang, Di Cui, Zheng-chao Nie, Bang-sheng Ding, Jiang-hua Cheng, Chuan-zhong Mei

**Affiliations:** 1 Division of Life Sciences and Medicine, Department of Clinical Laboratory, First Affiliated Hospital of University of Science and Technology of China, Hefei, Anhui, China; 2 School of Laboratory Medicine, Bengbu Medical College, Bengbu, Anhui, China; 3 Institute of Agro-products Processing Research, Anhui Academy of Agricultural Sciences, Hefei, Anhui, China

**Keywords:** Ureaplasma urealyticum, Mycoplasma hominis, Drug sensitivity

## Abstract

The objective of this study was to analyze the infection rate and drug resistance of *Ureaplasma urealyticum* (UU) and *Mycoplasma hominis* (MH) in the genitourinary tract of Chinese patients. From December 2018 to June 2019, vaginal secretion or urinary secretion of outpatients in our hospital were selected for culture and drug sensitivity analysis of *Ureaplasma urealyticum* and *Mycoplasma hominis*. In 4082 Chinese samples, 1567 Mycoplasma were detected, a detection rate of 38.39%, among which 1366 cases were UU single positive, accounting for 33.47%, 15 cases were MH single positive, accounting for 0.36%, 186 cases were UU and MH mixed positive, accounting for 4.56%. The most affected age groups were 21-30 years and 31-40 years, accounting for 19.09 and 15.05%, respectively. The results of drug sensitivity showed that doxycycline, minocycline, josamycin, clarithromycin, and roxithromycin were more sensitive to mycoplasma infection. The distribution of *Ureaplasma urealyticum* and *Mycoplasma hominis* in the human genitourinary system and their sensitivity to antibiotics is different for sex and age groups.

## Introduction

*Ureaplasma urealyticum* (UU) and *Mycoplasma hominis* (MH) are the most common Mycoplasma bacteria types, which are one of the main causes of male non-gonococcal urethritis, female cervical infection, abortion, and other diseases (1,2). In recent years, with the abuse of antibiotics, non-standard drug use, repeated infection, and other factors, the drug resistance of UU and MH has increased, which brings many difficulties to a standardized clinical treatment (3,4). In order to determine the infection rate and drug resistance of *Ureaplasma urealyticum* and *Mycoplasma hominis* in China, we examined the culture and drug sensitivity in 4082 cases of urogenital tract infection.

## Material and Methods

### General data

A total of 4082 patients with urogenital tract infection were selected from the Departments of Urology, Gynecology, Obstetrics, Reproductive Center, and Dermatovenereology of the First Affiliated Hospital of USTC from December 2018 to July 2019, including 1458 males and 2624 females, aged 34.53±12.42 years. The patients were initially diagnosed and did not use antibiotics within two weeks before sample collection. The study was approved by the Ethics Committee of the First Affiliated Hospital of USTC, and informed consent was signed by patients participating in the study.

### Specimen collection

For male patients, a sterile cotton ball was used to clean the urinary meatus, and a sterile cotton swab was then inserted with rotation into the urinary meatus for 1-2 cm to collect urethral secretion for immediate examination. For female patients, a sterile cotton swab was used to wipe the mucus on the cervical surface. Then, a special swab was inserted for 1-2 cm with rotation several times and remained for 15 s to collect a sample for examination.

### Culture, identification, and drug sensitivity of Mycoplasma

Mycoplasma IC kit produced by Zhuhai Lizhu Reagent Co., Ltd. (China) was used. All the reagents were used within the validity period. Drugs tested for sensitivity included: doxycycline, minocycline, josamycin, clarithromycin, roxithromycin, azithromycin, ofloxacin, levofloxacin, and sparfloxacin. High and low concentrations of each antibiotic were used simultaneously. The diluent was added to the 2.9-mL mark of the dry powder bottle of the culture medium, shaken well, and 50 μL was transferred to hole C after the dry powder was completely dissolved as the negative control. The swab was put into the culture solution, squeezed, and rotated to completely dissolve the sample. Then, 50 μL of the culture solution was placed into each hole, and 1-2 drops of liquid paraffin was placed into the remaining holes. The samples were incubated at 35-37°C for 24 or 48 h. UU results were observed after 24 h and MH results after 48 h. If the color of the culture hole changed from yellow to red, this indicated that there was Mycoplasma growth (+), if there was no color change (yellow), this indicated no Mycoplasma growth (-). The drug sensitivity was checked as follows: when the indicator hole indicated that there was growth of UU or MH and the high and low drug concentration pores did not turn red, the drug was considered sensitive (S); when the low concentration hole turned red and the high concentration hole remained unchanged, it was considered intermediary (I); when the high and low concentration holes turned red, it was considered resistant (R).

The SPSS 20.0 software (IBM, USA) was used for statistical analysis. Continuous variables of normal distribution are reported as means±SD, and the comparison between two groups was done with the *t*-test. Continuous variables of non-normal distribution are reported as median (interquartile range), and the comparison between the two groups was done with a non-parameter test. The counting data are reported as percentage (%), and chi-squared test was used for comparison between the two groups. The significance level was α=0.05.

## Results

### Infection rate of Mycoplasma

There were both single infections and mixed infections in the 4082 Chinese patients. The single infection rate of UU was 33.47% and of MH was 0.36%, and the mixed infection rate of UU+MH was 4.56%. The infection rate of Mycoplasma in females was 48.21%, among which the infection rates of UU, MH, and UU+MH were 41.65, 0.38, and 6.17%, respectively, and that of Mycoplasma in males was 20.71%, among which the infection rates of UU, MH, and UU+MH were 18.72, 0.34, and 1.65%, respectively. The infection rate of Mycoplasma and of UU infection in females was significantly higher than that in males (P<0.001). There was no significant difference between sexes for MH infection (P=0.847). The mixed infection rate of UU+MH in women was higher than that in men (P<0.001). See Table 1 for details.

**Table 1:** Detection rate of Mycoplasma in 4082 Chinese samples.

Gender	Number	UU (+) MH (-)	UU (-) MH (+)	UU (+) MH (+)	Total
Male	1458 (35.72)	273 (18.72)	5 (0.34)	24 (1.65)	302 (20.71)
Female	2624 (64.28)	1093 (26.77)	10 (0.25)	162 (3.97)	1265 (30.99)
Total	4082 (100.00)	1366 (33.47)	15 (0.36)	186 (4.56)	1567 (38.39)
χ^2^ value		734.682	0.037	44.179	299.579
P value		<0.001	0.8475	<0.001	<0.001

Data are reported as number and percent within parentheses. The chi-squared test was used for statistical analysis. UU: *Ureaplasma urealyticum*; MH: *Mycoplasma hominis*.

**Table 2:** Sensitivity to nine drugs of either/or *Ureaplasma urealyticum* and *Mycoplasma hominis*.

Drug	Sensitive (n, %)	Resistant (n, %)	Intermediate (n, %)
Doxycycline	1518 (96.88)	34 (2.17)	7 (0.45)
Minocycline	1522 (97.13)	24 (1.53)	21 (1.34)
Josamycin	1549 (98.85)	11 (0.70)	7 (0.45)
Clarithromycin	989 (63.12)	250 (15.95)	328 (20.93)
Roxithromycin	1325 (84.56)	203 (12.95)	39 (2.49)
Azithromycin	334 (21.31)	329 (21.00)	904 (57.69)
Ofloxacin	295 (18.83)	414 (26.42)	858 (54.75)
Levofloxacin	122 (7.79)	414 (26.42)	1031 (65.79)
Sparfloxacin	231 (14.74)	214 (13.66)	1122 (71.60)
Total	1567 (100.00)		

### Comparison of Mycoplasma detection in different age groups

There were 19 positive patients under 20 years old, with a detection rate of 0.48%; 779 positive patients (19.09%) between 21 and 30 years old; 615 positive patients (15.05%) between 31 and 40 years old, with a detection rate of 15.05%; 121 positive patients (2.96%) between 41 and 50 years old; and 33 positive patients (0.81%) over 50 years old. The total detection rate of each age group was the highest in the 21-30 and 31-40 age groups. The detection rate of the 21-30 age group was significantly higher than that of the ≤20, 41-50, and >50 age groups, 31-40 age group was higher than that of ≤20, 41-50, and >50 age groups, and there was no significant difference between 21-30 age group and 31-40 age group (χ^2^=1.591, P=0.207). See Supplementary Table S1 for details.

### Sensitivity results of nine drugs

The top three drug sensitivity rates of 1366 single UU-positive samples were josamycin (99.63%), minocycline (97.52%), and qianglimycin (97.29%); the top three drug resistance rates were ofloxacin (19.91%), levofloxacin (18.23%), and azithromycin (9.44%). The top three drug sensitivity rates of 15 single MH-positive samples were doxycycline (100%), minocycline (100%), and josamycin (100%); the top three drug resistance rates were clarithromycin (100%), roxithromycin (100%), and azithromycin (100%). The top three drug sensitivity rates of 186 UU- and MH-positive samples were minocycline (94%) doxycycline (93.55%), and josamycin (93.01%); the top three drug resistance rates were azithromycin (99.46%), clarithromycin (98.92%), and roxithromycin (97.31%). After summarizing the drug resistance of all 1567 Mycoplasma positive samples, we found that the sensitivity of doxycycline, minocycline, and josamycin exceeded 95%, while the drug resistance rate of ofloxacin, levofloxacin, and azithromycin exceeded 20%. See Supplementary Table S2 and Table 2 for details.

## Discussion

The Mycoplasma that can cause reproductive system infection mainly includes *Ureaplasma urealyticum* and *Mycoplasma hominis*. At present, these two Mycoplasma can cause pyelonephritis, vaginitis, pelvic inflammation, and other diseases, affecting the quality of life of patients. Therefore, once diagnosed, we must give timely and standardized treatment (5). The results of this study showed that the total infection rate of Mycoplasma in 4082 Chinese patients was 38.39%, mainly due to UU single infection, which was not consistent with the results of some research (6–8), indicating some differences in the distribution of Mycoplasma in different areas, which may be due to the use of antibiotics, sampling sites, detection reagents, and laboratory precision level in different areas.

The rate of Mycoplasma infection was 48.21% in women and 20.71% in men. The result is different from other areas in China and other countries (9–11). This may be due to the physiology of women, which is more favorable to infection by Mycoplasma. Another reason could be the number of infertility patients or women with other problems in the reproductive center, which has increased in recent years.

The higher infection rate in the 21-30-year and 31-40-year groups may be due to the fact that these age groups are in the childbearing period and have active sexual lives. Some patients had multiple sexual partners or other pathogens such as *Neisseria gonorrhoeae* and Chlamydia, and the vast majority of Mycoplasma infections are sexually transmitted. A promiscuous sexual life would also increase the probability of infection (12).

Mycoplasma have no cell wall and are naturally resistant to antibiotics acting on the cell wall such as β-lactams. Therefore, antibiotics acting on nucleoprotein should be selected to achieve effective treatment. In this study, doxycycline and minocycline were selected as tetracycline antibiotics, josamycin, clarithromycin, roxithromycin, and azithromycin as macrolides, and ofloxacin, levofloxacin, and sparfloxacin as quinolones. Our results showed that UU infection was sensitive to most antibiotics, such as josamycin, minocycline, doxycycline, and clarithromycin, and the drug resistance rate was low. MH infection and UU+MH mixed infection were resistant to clarithromycin, roxithromycin, azithromycin, ofloxacin, and levofloxacin. This is because MH has multiple drug resistance genes, natural resistance to most macrocyclic ester drugs, and cross-resistance to quinolones (13). In this study, the sensitivities of doxycycline, minocycline, and josamycin were over 95%, while the resistance of ofloxacin, levofloxacin, and azithromycin were over 20%, which indicated that appropriate antibiotics should be selected for different types of Mycoplasma.

In summary, clinicians should carry out routine Mycoplasma culture and identification and drug sensitivity tests to select appropriate antibiotics according to the results and achieve the best treatment effect.

## Supplementary Material

Click here to view [pdf].
